# Hospital and Staff Liability for Negligence

**Published:** 1893-03-25

**Authors:** 


### The Hospital

An Institutional Journal of

Science, Medicine, Nursing, and Philanthropy.

*For Hospitals, Asylums, Infirmaries, Dispensaries, Medical Practitioners, Students, Nurses, and the Charitable Public.*

Vol. XIII.—No. 339.] [March 25, 1893

### HOSPITAL AND STAFF LIABILITY FOR NEGLIGENCE.

Our attention has been called to this question by Dr. O'Reilley, of the Toronto General Hospital, in consequence of certain New York hospitals having been sued for the affirmed negligence of their staff. The question of the liability of the authorities of a hospital for the negligence of its staff has long since been set at rest in this country, and so far as we are able to judge it does not seem likely to be re-opened. We are accustomed to look upon the common law as the perfection of common sense, and with regard to this subject it certainly seems to be so.

There is little reason to doubt that the law on the subject in all civilized countries is substantially the same as in our own. But we observe with regret that of late years efforts have been frequently made to extend the liability not only of hospital authorities, but also of the staff. This appears to be particularly the case in the United States, where, probably owing to the differences which exist in the laws of each state, and also, no doubt, to the mixed character of the population, the settlement of legal questions is a much slower process than in an older country like our own. We have been unable to discover a single reported case in any of the recognized legal reports in this country of an attempt to fix on the authorities of a hospital a legal liability for the negligence of its staff. No such action could possibly be maintained without the plaintiff showing conclusively not only that the act or acts complained of were caused by negligent treatment on the part of the staff, but also that the authorities had been guilty of negligence in the selection and appointment of the staff. That being so, and taking into consideration the care bestowed in the selection of each individual member of the staff of every hospital throughout the country, it is perhaps not to be wondered at that few traces can be found of any such actions having been brought here. We envy the temerity of any one, even in these days when speculative actions are only too numerous, who would commence such an action. The immunity enjoyed by the staff of a hospital appears to be almost as complete as that of the management. After a most exhaustive search we have discovered but one case in which the skill and care of a member of the staff of a hospital has been impugned. In the year 1866 one Perionowsky, a name strangely suggestive of that latter-day creation, "the destitute alien," brought an action against two surgeons upon the staff of St. George's Hospital for alleged maltreatment by them in the administration of a hot-bath which they had ordered, but which it was no part of their duty personally to superintend, and at the actual administration of which they were not present. The late Lord Chief Justice Cockburn, before whom the action was tried, held that the plaintiff as a hospital patient was not entitled to expect more than the usual and ordinary degree of care and attention at the hands of the surgeons, and that if they were not personally cognizant of the alleged ill-usage they would not be liable. The jury found this to be so, and a verdict was at once entered for the defendants.

We venture to think that no better illustration can be afforded of the general excellence of our hospitals and kindred institutions than the fact that that case is the only one to be found in our legal reports. It would be well that the public should bear this in mind, and hesitate long before condemning hospitals or lending their ears to so called "hospital scandals." As we understand the law of the country, therefore, it seems to us to be clear that whatever may be the amount of negligence exhibited by a member of the staff of a hospital, the authorities of the institution are in no way liable so long as they have exercised due care and diligence in the selection and appointment of the member of the staff whose conduct is complained of. The only duty or obligation cast upon the members of the staff of a hospital individually is that referred to by the late Lord Chief Justice in the case we have quoted, viz., to exercise the usual and ordinary degree of care and attention which is to be expected of and attributable to physicians, surgeons, and others of like standing and experience.

With regard to the United States, we think that we shall be taken as fairly stating the law if we say that its principles are, in the main, in conformity with those of this country. The hospital authorities, if they have exercised due care in employing the members of the staff, will not be held liable for any injury which may be caused to a patient by the unskilful treatment of a member of such staff; and this, in spite of the fact that the hospital institutions of the United States receive aid directly from the Government, and are not voluntarily supported institutions, as is the case in England.

The members of the hospital staff in America are, however, on a somewhat different footing from that of their English brethren. They are held to be liable for gross negligence when their services are rendered gratuitously, although, if they receive remuneration, they will be treated on precisely similar grounds to those obtaining in England—that is to say, each member will be judged according to the skill and experience which he may reasonably be expected to possess and to exercise.

The decided cases bearing upon the matter under discussion are much more numerous in the United States than in this country, a fact which would seem to indicate that the American citizen considers the use of a hospital and its staff as a right rather than as a privilege. No doubt this attitude of the Americans is largely to be accounted for by the fact above referred to, that State support is liberally extended to the very large majority of hospitals in that country.

In conclusion, we wish to add a few words upon a question which has recently been raised in the United States and also in Canada. It has been asked why the authorities of a hospital where a member of its staff is sued for negligence should not recoup him for any costs and damages which he may incur in defending the action. With regard to this, there is much to be said on both sides. On the one hand, it might be urged, and very properly urged, that litigation by patients who considered themselves to have been carelessly treated would be extremely frequent, if it were known that the hospital acknowledged responsibility. It is quite possible also that the flow of subscriptions and donations might be impeded by a hospital adopting the course suggested.

But on the other hand, we are unable to see how the authorities of a hospital can be separated from its staff. We look upon the two as one corporate body standing together and fighting together for the public good, irrespective of race or religion, and as such (and until it can be shown that litigation is materially and unreasonably increased by such a course), we think that hospital authorities would be best advancing the cause of humanity without mentioning that of science, if they gave forth to the world that, being responsible to patients for the proper selection of each individual member of their respective staffs, they, in addition, were prepared to take upon themselves the defence of the conduct of each member while employed by them in their institution.

